# Cholera Epidemic — Lusaka, Zambia, October 2017–May 2018

**DOI:** 10.15585/mmwr.mm6719a5

**Published:** 2018-05-18

**Authors:** Nyambe Sinyange, Joan M. Brunkard, Nathan Kapata, Mazyanga Lucy Mazaba, Kunda G. Musonda, Raymond Hamoonga, Muzala Kapina, Fred Kapaya, Lwito Mutale, Ernest Kateule, Francis Nanzaluka, James Zulu, Chileshe Lukwesa Musyani, Alison V. Winstead, William W. Davis, Hammad S. N’cho, Nelia L. Mulambya, Patrick Sakubita, Orbie Chewe, Sulani Nyimbili, Ezinne V.C. Onwuekwe, Nedghie Adrien, Anna J. Blackstock, Travis W. Brown, Gordana Derado, Nancy Garrett, Sunkyung Kim, Sydney Hubbard, Amy M. Kahler, Warren Malambo, Eric Mintz, Jennifer Murphy, Rupa Narra, Gouthami G. Rao, Margaret A. Riggs, Nicole Weber, Ellen Yard, Khozya D. Zyambo, Nathan Bakyaita, Namani Monze, Kennedy Malama, Jabbin Mulwanda, Victor M. Mukonka

**Affiliations:** ^1^Ministry of Health, Lusaka, Zambia; ^2^Zambia National Public Health Institute, Lusaka; ^3^Zambia Field Epidemiology Training Program, Lusaka; ^4^CDC; ^5^Africa Centres for Disease Control and Prevention; ^6^World Health Organization; ^7^Copperbelt University, School of Medicine, Ndola, Zambia.

On October 6, 2017, an outbreak of cholera was declared in Zambia after laboratory confirmation of *Vibrio cholerae* O1, biotype El Tor, serotype Ogawa, from stool specimens from two patients with acute watery diarrhea. The two patients had gone to a clinic in Lusaka, the capital city, on October 4. Cholera cases increased rapidly, from several hundred cases in early December 2017 to approximately 2,000 by early January 2018 (Figure). In collaboration with partners, the Zambia Ministry of Health (MoH) launched a multifaceted public health response that included increased chlorination of the Lusaka municipal water supply, provision of emergency water supplies, water quality monitoring and testing, enhanced surveillance, epidemiologic investigations, a cholera vaccination campaign, aggressive case management and health care worker training, and laboratory testing of clinical samples. In late December 2017, a number of water-related preventive actions were initiated, including increasing chlorine levels throughout the city’s water distribution system and placing emergency tanks of chlorinated water in the most affected neighborhoods; cholera cases declined sharply in January 2018. During January 10–February 14, 2018, approximately 2 million doses of oral cholera vaccine were administered to Lusaka residents aged ≥1 year. However, in mid-March, heavy flooding and widespread water shortages occurred, leading to a resurgence of cholera. As of May 12, 2018, the outbreak had affected seven of the 10 provinces in Zambia, with 5,905 suspected cases and a case fatality rate (CFR) of 1.9%. Among the suspected cases, 5,414 (91.7%), including 98 deaths (CFR = 1.8%), occurred in Lusaka residents.

**FIGURE:**
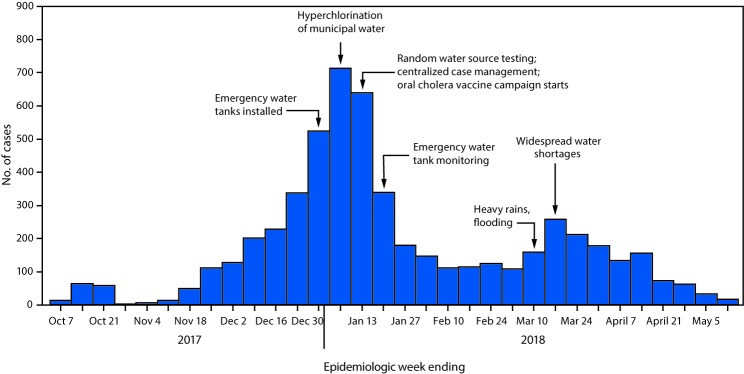
Number of reported cholera cases and related events, by week — Lusaka, Zambia, October 2017–May 2018

## Investigation and Results

MoH worked with multiple organizations, including the Zambia National Public Health Institute (ZNPHI), the Zambia Field Epidemiology Training Program (ZFETP), CDC, the World Health Organization (WHO), and the Africa Centres for Disease Control and Prevention (Africa CDC) to investigate the cholera outbreak and guide targeted, timely response activities. A suspected cholera case was defined as the development of acute watery or “rice water” diarrhea (three or more events within a 24-hour period) with or without vomiting or dehydration in any person of any age.* A confirmed case was defined as isolation of *V. cholerae* O1 from the stool of a person with suspected cholera.

**Epidemiologic investigations.** To assess knowledge, attitudes, and practices (KAP) regarding water, sanitation, and cholera, a cross-sectional survey was conducted in mid-December 2017 among 267 households in the most heavily affected neighborhoods of Lusaka. The KAP results indicated that most respondents (58%) believed poor hygiene to be the cause of cholera; 63% identified drinking contaminated water as a risk factor. To identify factors associated with transmission, a matched case-control study was conducted during December 18–21. Surveyors interviewed 81 case-patients with confirmed or suspected cholera and 130 controls. Preliminary results indicate that the odds of developing cholera were higher among those who had contact with a person with cholera (unadjusted odds ratio [OR] = 6.6; 95% confidence interval [CI] = 2.3–22.8) or who reported consumption of untreated water (OR = 3.6; 95% CI = 1.5–10.2) and were lower for females (OR = 0.3; 95% CI = 0.1–0.6). A total of 98 deaths occurred during October 4, 2017–May 12, 2018, in Lusaka; 40 (41%) deaths were reported by cholera treatment centers (CTCs), and 58 (59%) deaths occurred in the community. To identify risk factors associated with death from cholera, a case-control mortality study was conducted during January 12–March 26, 2018, with 32 cases (cholera deaths) and 64 controls (cholera survivors) matched by age and date of onset. Preliminary results indicated that the odds of dying from cholera were less among those who stayed an additional night at a CTC (OR = 0.30; 95% CI = 0.04–0.88), underscoring the importance of access to cholera treatment, including rehydration.

**Case management training**. Based on health care worker surveys and CTC assessments, ZNPHI, CDC, and the District Health Management Team organized cholera training for health care workers to help them address knowledge gaps. Training focused on cholera detection and clinical management and reached approximately 100 health care workers in Lusaka during January–February 2018.

**Water source testing and monitoring.** MoH, ZNPHI, and CDC, in collaboration with the Lusaka Water and Sewerage Company (LWSC), implemented a water source monitoring program to test randomly selected drinking water sources for free chlorine residual and *Escherichia coli* (*E. coli),* an indicator of fecal contamination. Approximately 220 randomly selected water sources in areas across Lusaka affected by and not affected by cholera were tested in January 2018; results were provided daily to MoH, NPHI, and LWSC. In total, 160 (73%) of the 220 drinking water sources tested had inadequate levels of free chlorine residual (<0.2 mg/L); 41 (31%) of the 160 water sources with inadequate free chlorine residual were positive for *E. coli*. The most commonly contaminated water sources were shallow wells (91%) and boreholes (34%). On January 15, 2018, a daily monitoring program was begun for all emergency water tanks across Lusaka. Daily reports continue to be provided to LWSC and MoH regarding the locations of tanks that are empty and those that have free chlorine residual <1.0 mg/L^†^ so that immediate corrective action can be taken to refill tanks or boost chlorination in water trucks at the filling reservoirs.

**Clinical isolate characterization**. Of 2,054 stool specimens tested during October 4, 2017–May 12, 2018, at the University Teaching Hospital national reference laboratory in Zambia, 925 (45%) yielded *Vibrio cholerae* O1. The majority were serotype Ogawa; five isolates yielded *Vibrio cholerae* O1, serotype Inaba. Antibiotic susceptibility testing was conducted in mid-January 2018.^§^ All 50 isolates tested were sensitive to cotrimoxazole, tetracycline, chloramphenicol, and azithromycin; 72% were sensitive and 28% had intermediate sensitivity (i.e., response rates might be lower) to ampicillin. The first-line treatment for cholera cases was doxycycline for adults and cotrimoxazole for children.

## Public Health Response

In October 2017, MoH activated a national emergency operations center, using an incident management system to collaborate with other government ministries and partner organizations, including CDC, Africa CDC, the United Nations Children’s Fund (UNICEF), WHO, Zambia Red Cross, Médecins Sans Frontières, and others. To improve safe water supply, 282 emergency chlorinated water tanks were installed beginning in December 2017. In addition, household water treatment products were distributed to approximately 1 million households in the most affected areas. To strengthen surveillance, MoH disseminated information and trained staff members on standardized cholera case definitions and communication strategies and produced daily situation and outbreak reports. Cholera prevention and water treatment materials were developed and publicized through door-to-door campaigns, mass media, and by community health workers. A 2-dose oral cholera vaccine campaign was launched in cholera-affected subdistricts of Lusaka in January 2018. Approximately 1 million residents received 2 doses of oral cholera vaccine, representing approximately 80% of the targeted population and 50% of Lusaka’s population.

## Discussion

Cholera remains a global public health challenge, with an estimated 2.9 million cases occurring each year in countries with endemic disease and 1.3 billion persons at risk for infection (*1*). Large, rapidly escalating outbreaks of cholera are transmitted primarily through contaminated drinking water supplies. Cholera incidence can be reduced through increased access to safe water, sanitation, and hygiene (WASH) facilities and through behavioral changes resulting from community education and training; oral cholera vaccines are increasingly used as a temporizing measure. Since 1970, cholera has become endemic in many sub-Saharan African countries and remains a recurring major public health problem (*2*); recent outbreaks have occurred in Angola, the Democratic Republic of the Congo, Malawi, Tanzania, and Zimbabwe (*3*,*4*). Zambia’s first cholera outbreak was reported during 1977–1978, and outbreaks with approximately 11,000 cases occurred in 1991, 1992, and 1999 (*5*). The current outbreak has been concentrated in peri-urban areas of Lusaka, which have limited access to municipal water supplies or sewer systems, and where approximately 60% of Lusaka’s population resides (*6*).

Early case investigation indicated that consumption of contaminated water and contact with a person ill with cholera were likely risk factors for transmission, a hypothesis supported by findings from a case-control study and water source testing results. This information led to improvements in water supply and increases in chlorine levels throughout the municipal water distribution system, installation of emergency chlorinated water tanks in the communities at most risk, and widespread distribution of household water treatment products. Implementation of these activities was followed by a sharp reduction in cholera cases; however, the onset of a late rainy season and underlying WASH vulnerabilities, including water shortages resulting from emergency repairs of the city’s primary water treatment plant, led to a resurgence of cases in March 2018. Most areas affected by flooding have a high concentration of pit latrines and shallow wells, a situation conducive to contamination of drinking water sources. Heavy rainfall has been associated with previous cholera outbreaks in Zambia and across Africa (*7*,*8*).

In the current outbreak, a higher percentage of deaths occurred in the community (59%) than in CTCs (41%). Delay in seeking care is a known risk factor for cholera mortality but is most often associated with outbreaks in rural areas where transportation and distance to care are limiting factors (*9*). Preliminary qualitative data and community reports indicate that stigma over concern about being associated with poor hygiene might have played a role in patients delaying seeking care in Lusaka; findings from the KAP survey indicated that residents associated cholera with poor hygiene.

The findings from this outbreak investigation demonstrate the need for a robust public health response during the initial stages of a cholera outbreak and the importance of enhanced surveillance and continual efforts to maintain an adequate, chlorinated drinking water supply to achieve sustained outbreak control. However, cholera resurgence remains a risk unless underlying WASH vulnerabilities, including lack of access to safe drinking water and adequate sanitation, are addressed. The Global Task Force for Cholera Control recently proposed a comprehensive, multisectoral approach to reducing cholera deaths and ending local cholera transmission through proactive investments in preparedness, WASH, and oral cholera vaccine in known areas of cholera transmission (*10*). A resolution to support this approach will be considered at the World Health Assembly during May 2018.

**Summary**

**What is already known about this topic?**

Approximately 2.9 million cholera cases occur each year worldwide, and 1.3 billion persons are at risk for infection, usually from contaminated drinking water.

**What is added by this report?**

A cholera outbreak that began in October 2017 in Zambia has resulted in approximately 5,900 cases and 114 deaths. The government improved the water supply and administered oral cholera vaccine, but flooding led to a resurgence of cholera. A multisectoral and well-coordinated response was key to the control of the outbreak.

**What are the implications for public health practice?**

Gains can be made in outbreak control with a robust public health response. However, cholera resurgence remains a risk unless access to safe drinking water and adequate sanitation are assured.

## Footnotes

* WHO updated cholera case definition: http://www.who.int/cholera/task_force/GTFCC-Laboratory-support-public-health-surveillance.pdf.

† WHO recommends maintaining the following chlorine residuals during a cholera outbreak: 0.5 mg/L at all points in a piped supply, 1.0 mg/L at standposts and wells, and 2.0 mg/L in tanker trucks at filling.

§ Antibiotic susceptibility testing was conducted using the Bauer-Kirby disc diffusion method, and minimum inhibitory concentration (MIC) for azithromycin using the E-test according to Clinical and Laboratory Standards Institute guidelines. http://www.facm.ucl.ac.be/intranet/CLSI/CLSI-2017-M100-S27.pdf.
